# The Integral Membrane Protein ZMPSTE24 Protects Cells from SARS-CoV-2 Spike-Mediated Pseudovirus Infection and Syncytia Formation

**DOI:** 10.1128/mbio.02543-22

**Published:** 2022-10-05

**Authors:** Khurts Shilagardi, Eric D. Spear, Rachy Abraham, Diane E. Griffin, Susan Michaelis

**Affiliations:** a Department of Cell Biology, Johns Hopkins School of Medicine, Baltimore, Maryland, USA; b W. Harry Feinstone Department of Molecular Microbiology and Immunology, Bloomberg School of Public Health, Johns Hopkins University, Baltimore, Maryland, USA; Washington University School of Medicine

**Keywords:** COVID-19, SARS-CoV-2, ZMPSTE24, coronavirus, innate immune response, prelamin A, proteases, restriction factor

## Abstract

COVID-19 pandemic caused by severe acute respiratory syndrome coronavirus 2 (SARS-CoV-2) has had a devastating impact on global public health, emphasizing the importance of understanding innate immune mechanisms and cellular restriction factors that cells can harness to fight viral infections. The multimembrane-spanning zinc metalloprotease ZMPSTE24 is one such restriction factor. ZMPSTE24 has a well-characterized proteolytic role in the maturation of prelamin A, precursor of the nuclear scaffold protein lamin A. An apparently unrelated role for ZMPSTE24 in viral defense involves its interaction with the interferon-inducible membrane proteins (IFITMs), which block virus-host cell fusion by rigidifying cellular membranes and thereby prevent viral infection. ZMPSTE24, like the IFITMs, defends cells against a broad spectrum of enveloped viruses. However, its ability to protect against coronaviruses has never been examined. Here, we show that overexpression of ZMPSTE24 reduces the efficiency of cellular infection by SARS-CoV-2 Spike-pseudotyped lentivirus and that genetic knockout or small interfering RNA-mediated knockdown of endogenous ZMPSTE24 enhances infectivity. We further demonstrate a protective role for ZMPSTE24 in a Spike-ACE2-dependent cell-cell fusion assay. In both assays, a catalytic dead version of ZMPSTE24 is equally as protective as the wild-type protein, indicating that ZMPSTE24’s proteolytic activity is not required for defense against SARS-CoV-2. Finally, we demonstrate by plaque assays that *Zmpste24^−/−^* mouse cells show enhanced infection by a genuine coronavirus, mouse hepatitis virus (MHV). This study extends the range of viral protection afforded by ZMPSTE24 to include coronaviruses and suggests that targeting ZMPSTE24’s mechanism of viral defense could have therapeutic benefit.

## INTRODUCTION

The COVID-19 pandemic, caused by SARS-CoV-2, has been the most challenging public health crisis in over a century. Although several therapeutic treatments are available, a better understanding of innate host cell restriction factors that can prevent viral infection could provide critical information for developing broad-spectrum therapeutics to prevent or treat COVID-19, as well as other emergent or established viral diseases, for instance, those caused by Zika, Ebola, influenza, HIV or dengue viruses (1–6). One such viral restriction factor, the integral membrane zinc metalloprotease ZMPSTE24, is the subject of this study.

ZMPSTE24 has a well-established role unrelated to viral defense. ZMPSTE24 is critical for human health and longevity through mediating the final step in proteolytic maturation of prelamin A, the farnesylated precursor of the nuclear scaffold protein lamin A encoded by *LMNA* (7–10). ZMPSTE24 cleaves the C-terminal 15 amino acids of prelamin A to generate mature lamin A. Mutations in the genes encoding either ZMPSTE24 or prelamin A that block this cleavage step cause premature aging diseases that include Hutchinson-Gilford progeria syndrome, mandibuloacral dysplasia, and restrictive dermopathy, which result from the accumulation of uncleaved farnesylated prelamin A (11–16). ZMPSTE24 has 7 transmembrane spans, forms an unusual barrel-like structure in the membrane with its zinc metalloprotease catalytic domain facing the inside of the barrel, and a side opening to admit substrates (9, 17–19). It contains a C-terminal KxxKxx endoplasmic reticulum (ER) retrieval motif and dually localizes to the ER and inner nuclear membrane (INM) (20). Although the sole known defined cellular role for ZMPSTE24 is the maturation of prelamin A, genetic studies on the yeast homolog Ste24 from our group and others have hinted at additional, albeit less-well-defined, roles for ZMPSTE24 in cellular processes that broadly converge on protein quality control and membrane stress. These processes include ER-associated degradation, the unfolded protein response, protein secretion, determination of membrane protein topology, and clearance of clogged translocons (21–28).

Intriguingly, ZMPSTE24 was also unexpectedly discovered to have a role in viral protection in a compelling study by M. E. Dorf and coworkers (29). Coimmunoprecipitation experiments identified ZMPSTE24 as an interactor with members of the interferon-induced transmembrane (IFITM) protein family, including IFITM1, IFITM2, and IFITM3 (29, 30). The IFITMs are well-characterized broad-spectrum viral restriction factors that are induced by interferon and can block the entry of many enveloped viruses (3, 5, 6, 31–35). IFITMs act at the hemifusion step of virus-host cell fusion by a mechanism that appears to involve “rigidifying” membranes, affecting membrane curvature in a way that precludes viral fusion (36–40). IFITM family members are small transmembrane proteins (125 to 133 amino acids) that localize to the plasma membrane (IFITM1) or endosomes (IFITM2 and -3) and inhibit viral entry at those sites (37, 41, 42). In general, overexpression of IFITMs protects cells from viral infection, while diminished IFITM expression increases cell susceptibility to infection. However, in some cases, IFITMs have been reported to facilitate virus-host cell fusion (43–46) (see Discussion).

Dorf’s group showed that, like the IFITMs with which it interacts, overexpression of ZMPSTE24 robustly protects cells from infection by many pathogenic enveloped viruses, but not all (e.g., murine leukemia virus [MLV] is an exception), and that its proteolytic activity is not needed in this role (29, 30). Furthermore, knockout mutations of *ZMPSTE24* (or small interfering RNA [siRNA] knockdown) in cells and mice caused them to succumb to infection by influenza A virus (IAV) and other viruses. Notably, in the absence of ZMPSTE24, IFITMs did not exert their protective effect and in cells lacking IFITMs, ZMPSTE24 had antiviral activity on its own. These findings led the authors to suggest that ZMPSTE24 may be a downstream effector of IFITMs (29, 30). While the mechanistic relationship between IFITMs and ZMPSTE24 remains to be clearly defined, these findings place ZMPSTE24 at an important position in the cell’s first line of innate defense against infection by many viruses.

Whether ZMPSTE24’s protective effect extends to coronaviruses has not yet been examined. Here, we determined that ZMPSTE24 defends cells from infection mediated by SARS-CoV-2 Spike protein using pseudovirus and cell-cell fusion assays. Furthermore, we demonstrated that ZMPSTE24’s catalytic activity is dispensable in this role and that a catalytically dead ZMPSTE24 mutant protein is more effective than its wild-type counterpart in coimmunoprecipitating IFITM3. We also showed that lack of ZMPSTE24 sensitizes mouse cells to infection with the coronavirus mouse hepatitis virus (MHV), thereby highlighting ZMPSTE24’s antiviral role in a genuine viral infection assay, thus extending its range of protection to include coronaviruses and motivating future work to decipher ZMPSTE24’s mechanism of viral restriction.

## RESULTS

### *ZMPSTE24* knockout cells are more susceptible than wild-type cells to infection by VSV-G-pseudotyped lentivirus.

**FIG 1:**
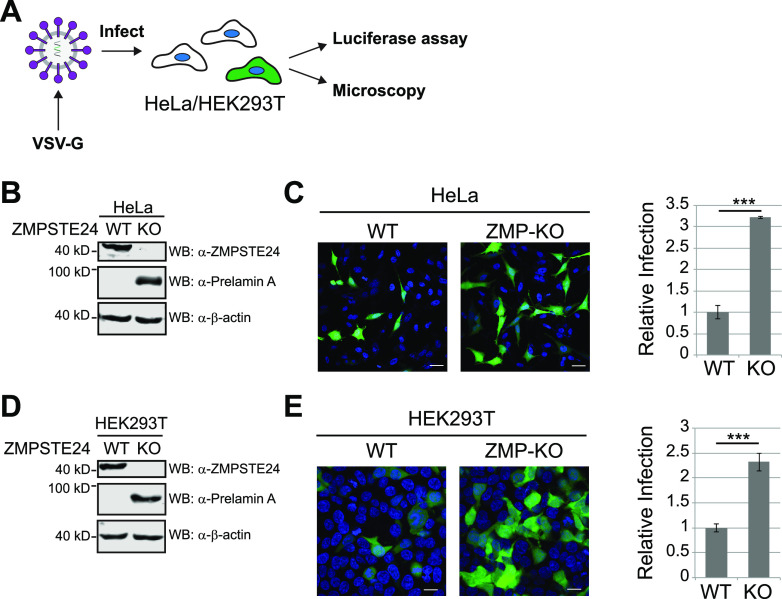
ZMPSTE24 deficiency sensitizes cells to VSV-G-pseudotyped lentivirus infection. (A) Schematic representation of the infection assay. HEK293T cells were exposed for 48 h to VSV-G-pseudotyped lentivirus encoding the reporter genes firefly luciferase and ZsGreen. Fluorescence microscopy detected Zsgreen-positive infected cells, and the luciferase activity in lysates provided a quantitative measure of infection. (B and D) Western blot confirming the presence or absence of ZMPSTE24 in HeLa and HEK293T cells used in this study. ZMPSTE24 was present in wild-type cells (ZMP-WT) and absent in cells in which ZMPSTE24 was knocked out (ZMP-KO) by CRISPR-Cas9. Correspondingly and as expected, prelamin A accumulated in ZMPSTE24 KO cells. In WT cells, ZMPSTE24 cleaved off the C-terminal 15 amino acids of prelamin A to yield mature lamin A, and so essentially no prelamin A was present. The rat anti-prelamin A antibody used here uniquely recognizes the 15 C-terminal residues of prelamin A, which are retained when ZMPSTE24 is absent. (C and E) At left are representative confocal fluorescence microscopy images of WT and ZMP-KO HeLa and HEK293T cells, respectively, 48 h postinfection (MOI of 0.1). ZsGreen-positive cells indicate infected cells; DAPI stained all cell nuclei. Scale bars, 20 μm. At right is quantification of the relative fold efficiency of infection for ZMPSTE24 WT and KO cells, based on the luciferase activity, with the activity for WT cells normalized to 1.0. Standard deviation of the mean was calculated from 3 independent experiments. Statistical significance was determined by unpaired, two-tailed *t* test. ns, *P* > 0.05; *, *P* < 0.05; **, *P* < 0.01; ***, *P* < 0.001; ****, *P* < 0.0001.

As a starting point in this study and to verify the published results for the protective role of ZMPSTE24 in human cell lines, we produced lentivirus pseudotyped with vesicular stomatitis virus glycoprotein (VSV-G) that carried two reporter genes, ZsGreen to visualize infected cells by fluorescence microscopy and luciferase to quantitate infection (Fig. 1A). Cell lines (HeLa and HEK293T) in which CRISPR/Cas9 genome editing had been used to generate *ZMPSTE24* knockout (KO) alleles were validated for the absence of the ZMPSTE24 protein (and also the expected accumulation of prelamin A due to retention of its C-terminal 15 amino acids, which are recognized by the prelamin A antibody) (Fig. 1B and D). We infected wild-type (WT) and *ZMPSTE24* KO HeLa and HEK293T cells with VSV-G-pseudotyped lentivirus, which uses endogenous low-density lipoprotein receptor as its entry receptor. For both cell types, *ZMPSTE24* KO cells show a substantially greater level of infection than WT cells by fluorescence microscopy (Fig. 1C and E). Quantitation of the luciferase signal confirmed these higher levels of infection in *ZMPSTE24* KO versus WT cells, which were ~3-fold higher for HeLa cells and ≥2-fold higher for HEK293T cells. Thus, in agreement with previous findings (29), *ZMPSTE24* deficiency sensitizes cells to infection by VSV-G-pseudotyped lentivirus.

### Depletion of ZMPSTE24 enhances infection by SARS-CoV-2 Spike-pseudotyped lentivirus.

**FIG 2:**
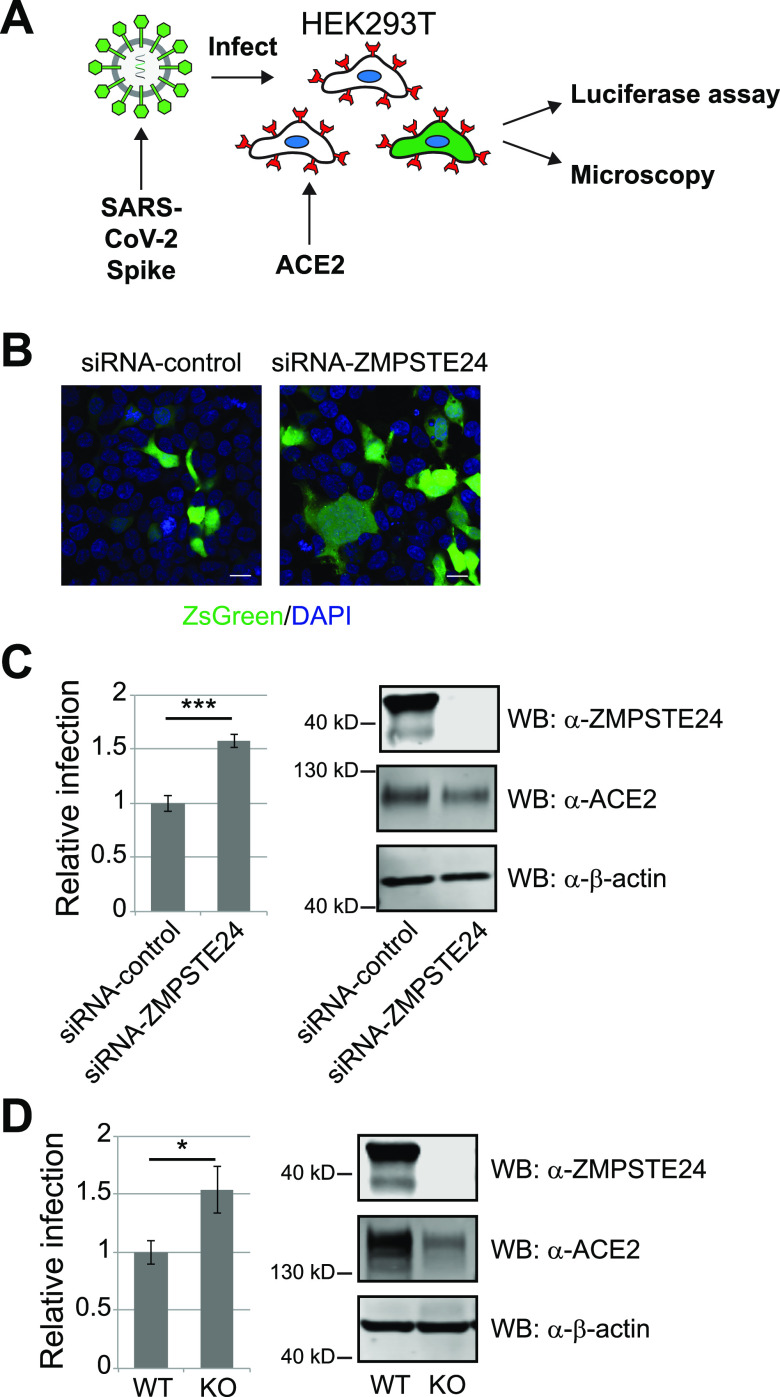
ZMPSTE24 deficiency sensitizes cells to SARS-CoV-2 S-pseudotyped lentivirus infection. (A) Schematic of the infection assay. SARS-CoV-2 Spike-pseudotyped lentivirus (the green hexagon is Spike) encodes luciferase and the ZsGreen reporter. HEK293T cells express ACE2 (red cup shape). (B) HEK293T cells stably transduced with human ACE2 were transfected with control (scrambled) siRNA or siRNA targeting *ZMPSTE24* for 36 h, followed by infection with Spike-pseudotyped virus at an MOI of 0.1 for 48 h. Cells were fixed, and representative confocal microscopy images are shown. ZsGreen-positive cells indicate infected cells; DAPI stained all cell nuclei. Scale bars, 10 μm. (C) Relative infection level was measured by the luciferase assay and normalized to the control siRNA. The Western blot confirmed siRNA knockdown of ZMPSTE24 and expression of ACE2. (D) WT and ZMPSTE24-KO HEK293T cells stably expressing ACE2-mCherry were infected with S-pseudotyped virus at an MOI of 0.1 for 48 h, and relative infection levels were measured by luciferase assay. The corresponding Western blot is shown. It should be noted in panels C and D that ZMPSTE24 (predicted mass of ~55 kDa) standardly migrates anomalously in SDS-PAGE at ~40 to 50 kDa (as a doublet [here], smear [as in Fig. 5C], or a singlet [as in Fig. 1]). Aberrant migration is a common feature of membrane proteins that can be seen for ZMPSTE24 here and elsewhere (10, 15, 17). Statistics were based on 3 independent experiments, as described for Fig. 1.

We generated SARS-CoV-2 Spike-pseudotyped lentivirus carrying ZsGreen and luciferase reporters to determine if ZMPSTE24 depletion influenced infection of HEK293T cells that had been stably transduced with *ACE2* (or *ACE2-mCherry*), which encodes the SARS-CoV-2 receptor (Fig. 2A). Knockdown (KD) of *ZMPSTE24* expression with siRNA resulted in increased infection by SARS-CoV-2 Spike-pseudotyped lentivirus compared to treatment with a control (scrambled) siRNA, as detected by immunofluorescence (Fig. 2B) and measured by the luciferase assay (Fig. 2C). Likewise, comparing infection of *ZMPSTE24* KO and WT cells, the lack of *ZMPSTE24* in KO cells increased infection, based on the luciferase assay (Fig. 2D). We note that the susceptibility of ZMPSTE24 KO cells to infection may actually be underestimated here due to the lower ACE2-mCherry levels in these cells compared to WT cells. Together, these data indicate that a diminished level of *ZMPSTE24* results in increased sensitivity to SARS-CoV-2 Spike pseudovirus infection, thus extending the reach of ZMPSTE24’s protective activity to include a coronavirus-pseudotyped virus.

### Overexpression of *ZMPSTE24* protects against SARS-CoV-2 pseudovirus infection.

**FIG 3:**
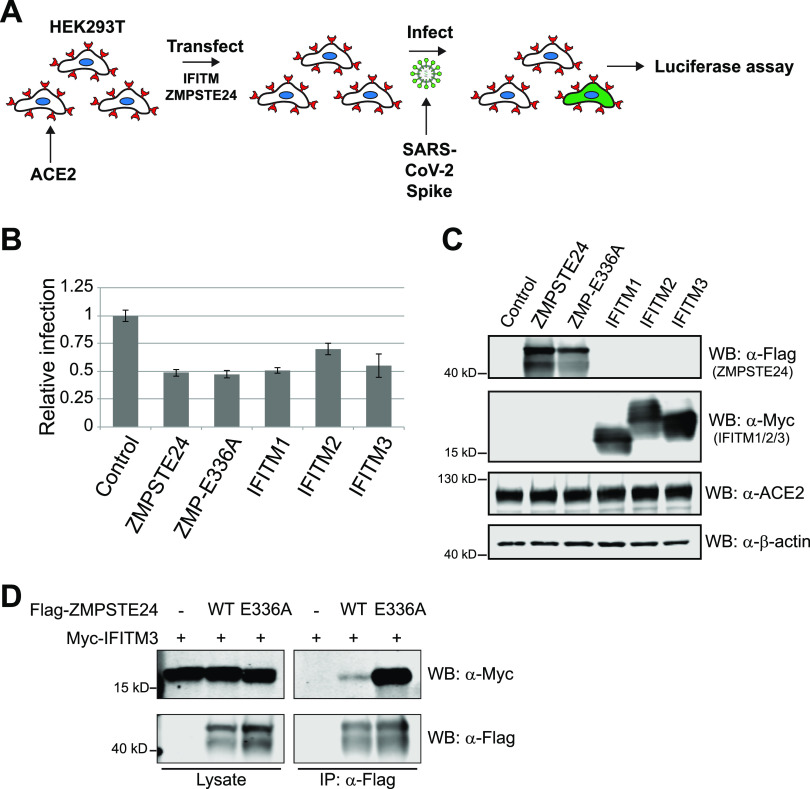
Overexpression of ZMPSTE24 protects cells against SARS-CoV-2 S-pseudotyped lentivirus infection. (A) Schematic of the infection assay. HEK293T cells stably expressing human ACE2 were transfected with plasmids encoding Flag-ZMPSTE24 (pSM3914), Flag-ZMPSTE24-E336A (a catalytically inactive version of ZMPSTE24) (pSM3915), Myc-IFITM1, -2, or -3 (pSM3873, pSM3874, or pSM3867, respectively), or empty vector (control) (pSM3978) for 36 h, then infected with S-pseudotyped virus at an MOI of 0.1 for 48 h and lysed, and luciferase was assayed. (B) Relative luciferase activities reflect infection efficiency, compared to the control (empty vector), set at 1. (C) Western blot showing levels of protein from the transfected constructs, ACE2, and β-actin as a loading control. Statistics were based on 3 independent experiments as described for Fig. 1. (D) Western blot showing that a catalytically dead version of ZMPSTE24 interacts with IFITM3 more strongly than WT ZMPSTE24. HEK293T cells were cotransfected with Myc-IFITM3 and empty vector, a plasmid that expressed Flag-ZMPSTE24, or a plasmid that expressed a catalytically dead version of the protease Flag-ZMPSTE24-E336A. After 36 h, cells were lysed in buffer containing 1% Triton X-100, as described in Materials and Methods, and proteins were immunoprecipitated with anti-FLAG magnetic beads for 2 h at 4°C. Immunoprecipitated proteins were resolved by SDS-PAGE, and ZMPSTE24 and IFITM3 proteins were detected by Western blotting using anti-FLAG and anti-Myc antibodies, respectively. The input lysate before coimmunoprecipitation is on the left; the right panel shows the coimmunoprecipitation and represents ~20-fold more sample than input.

The overexpression of IFITM1, -2 or -3 protects cells from infection by SARS-CoV-1 and SARS-CoV-2 (44, 45, 47, 48). Given that ZMPSTE24 interacts with the IFITMs (29, 30) (see also Fig. 3D), we also asked whether overexpression of *ZMPSTE24* would provide greater protection against SARS-CoV-2 pseudovirus infection than that conferred by endogenous ZMPSTE24. HEK293T cells stably expressing ACE2 were transiently transfected with a control (vector-only) plasmid or plasmids overexpressing N-terminally Myc-tagged IFITMs (Myc-IFITM1-3), N-terminally Flag-tagged ZMPSTE24 (ZMPSTE24), or a catalytically inactive mutant form (ZMPSTE24-E336A) and then exposed to SARS-CoV-2-pseudotyped lentivirus (Fig. 3A). The results are shown in Fig. 3B and C. As expected based on previously published work, overexpressed IFITM1, -2, and -3 all protected cells significantly (decreasing infection to 50% to 75% of that with vector only) (44, 45). Notably, the overexpression of ZMPSTE24 also decreased SARS-CoV-2 pseudovirus infection to a similar level as that seen for overexpression of the IFITMs. Furthermore, the ZMPSTE24-E336A catalytically dead mutant inhibited infection roughly to the same extent as WT ZMPSTE24, in agreement with previous findings with other viruses and pseudoviruses (29). These findings established that ZMPSTE24 overexpression, like that of the IFITMs, protects against SARS-CoV-2 pseudovirus infection and, furthermore, that its catalytic activity is not necessary for protection.

We also performed coimmunoprecipitation experiments between Flag-ZMPSTE24 and Myc-IFITM3. Interestingly, we found that the catalytically dead version of the protease, ZMPSTE24-E336A (15, 19), is far more effective than WT ZMPSTE24 at pulling down IFITM3 (Fig. 3D). We discuss possible explanations for this finding in the Discussion. It should be noted, however, that despite better binding to IFITM3, the catalytically inactive ZMPSTE24 does not afford better protection than WT ZMPSTE24 to SARS-CoV-2 pseudovirus infection (Fig. 3C).

### SARS-CoV2 Spike-mediated cell-to-cell fusion is enhanced by ZMPSTE24 deficiency and inhibited by ZMPSTE24 overexpression.

**FIG 4:**
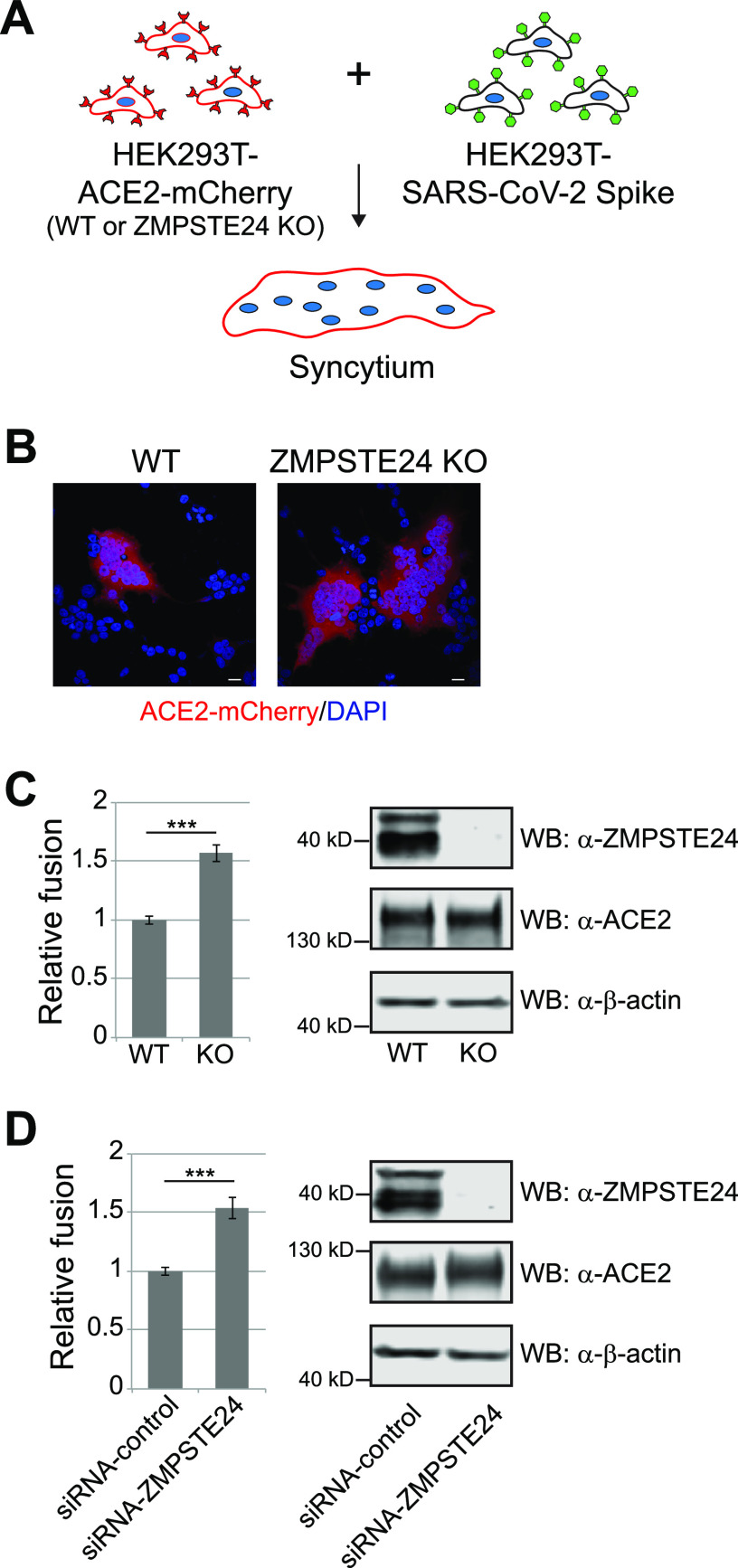
ZMPSTE24 deficiency sensitizes cells to SARS-COV-2 Spike-induced syncytia formation. (A) Schematic representation of the cell fusion assay. HEK293T “recipient” cell lines (WT or *ZMPSTE24*-KO) stably expressing ACE2-mCherry were mixed with an equal number of HEK293T “donor” cells transiently transfected with SARS-CoV-2 Spike protein. The mixed cell population was allowed to fuse for 3 h, fixed, and imaged by confocal microscopy. (B) Representative confocal images of the mixed cell population, with syncytia evident when recipient cells were deficient in ZMPSTE24. Fused cells appear as large multinucleated (DAPI, blue) syncytia labeled in red (ACE2-mCherry). Scale bars, 20 μm. (C) Quantification of the cell fusion experiments in panel B. The cell fusion efficiency was calculated as a percentage of nuclei in syncytia, compared to the total number of the nuclei on a given 40× microscopic field, with WT set to 1. Five microscopic fields from 3 independent experiments were counted to calculate standard deviations (*n* = 15). (D) HEK293T cells stably expressing ACE2 were transfected with either control (scrambled) siRNA or siRNA targeting ZMPSTE24. After 36 h, cells were mixed with SARS-CoV-2 Spike-expressing cells to allow syncytia formation, and fusion was quantitated as for panel C. For panels C and D, the adjacent Western blots confirmed the absence of ZMPSTE24 protein in knockout and knockdown cells and roughly equal amounts of ACE2 or ACE2-mCherry receptor between cell lines. β-Actin served as a loading control.

To assess ZMPSTE24’s protective function in an assay independent of pseudovirus infection, we measured syncytia formation, also referred to as cell-to-cell fusion. Syncytia are large multinucleated cells resulting from the fusion of two or more cells and can occur by mixing two populations of cells, one expressing a viral fusion protein and the other expressing its cognate receptor (49, 50). In our assay, we mixed WT cells expressing SARS-CoV-2 Spike protein (“donor cells”) with cells (WT or *ZMPSTE24* KO) stably expressing ACE2-mCherry (“recipient cells”) (Fig. 4A). After coculture for 3 h, multinucleated syncytia could be observed by fluorescence microscopy (Fig. 4B), and the fraction of total nuclei present in syncytia was counted to determine the fusion index (Fig. 4C). When the recipient cells were ZMPSTE24 deficient (ZMPST24 KO) compared to WT, syncytia were more frequent (Fig. 4B, compare right and left panels) and had a higher fusion index (Fig. 4C), indicating that the lack of endogenous *ZMPSTE24* sensitized cells to SARS-CoV-2 Spike-mediated fusion.

We also performed this cell-to-cell fusion assay using siRNA to decrease ZMPSTE24 expression. In this case, WT recipient cells stably expressing ACE2 were first transfected with either scrambled control siRNA (WT) or siRNA targeting ZMPSTE24, followed by coculture and determination of the fraction of nuclei found in syncytia (Fig. 4D). Consistent with the findings using ZMPSTE24 KO cells, siRNA-mediated ZMPSTE24 knockdown enhanced the cell fusion index, compared to cells treated with the control siRNA (WT) that expressed the normal level of endogenous *ZMPSTE24*. Together, these results indicate that ZMPSTE24 deficiency enhances SARS-CoV-2 Spike-mediated syncytia formation, suggesting that endogenous ZMPSTE24 acts as a fusion inhibitor.

### ZMPSTE24 overexpression inhibits cell-to-cell fusion.

**FIG 5:**
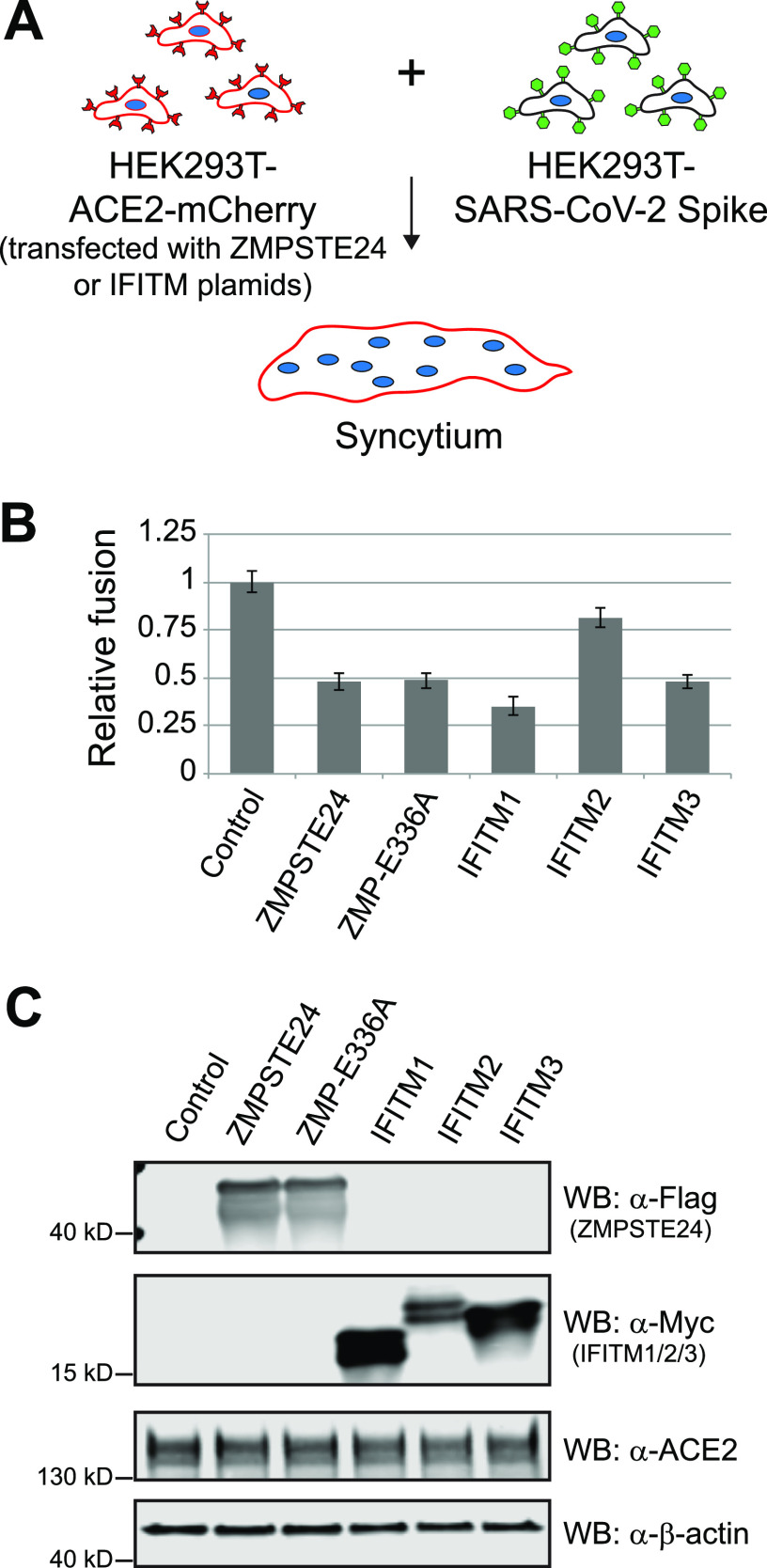
ZMPSTE24 overexpression inhibits Spike-mediated syncytia formation. (A) Schematic of cell fusion assay. HEK293T cells stably expressing ACE2-mCherry were transiently transfected with ZMPSTE24- or IFITM-encoding plasmids or empty vector (control) for 36 h prior to mixing with HEK293T cells expressing SARS-CoV-2 Spike protein, as in Fig. 4. (B and C) Quantification of syncytia formed and Western blots from transfected cells. ZMPSTE24 and catalytically dead ZMPSTE24-E336A were detected with anti-FLAG antibodies; IFITM1, IFITM2, and IFITM3 were detected with anti-Myc antibodies. Antibodies against ACE2 and β-actin confirmed equivalent amounts of ACE2-mCherry proteins between cell lines.

We also tested whether overexpression of ZMPSTE24 inhibited SARS-CoV-2- Spike-mediated syncytia formation, as was recently shown for the IFITMs (45, 49, 51). To do so, we transfected ACE2-mCherry-expressing recipient cells with plasmids that expressed WT ZMPSTE24, the catalytically dead mutant ZMPSTE24-E336A, IFITMs1 to - 3, or a vector-only control, and assessed their fusion efficiency with donor cells expressing SARS-CoV-2 Spike protein (Fig. 5A). In agreement with published results, overexpression of any of the IFITMs inhibited fusion, with IFITM1 having the most pronounced effect (Fig. 5B). Notably, overexpression of ZMPSTE24 also showed a strong inhibitory effect, and consistent with the pseudovirus studies, the catalytically dead mutant ZMPSTE24-E336A also inhibited cell fusion. Equivalent expression levels of IFITMs and the WT and mutant forms of ZMPSTE24 were validated by Western analysis (Fig. 5C).

Together, the results from the cell fusion assays in Fig. 4 and 5 indicate that ZMPSTE24 inhibits SARS-CoV-2 Spike-mediated cell fusion in a dose-dependent manner, similar to what has been shown for the IFITMs. Fusion was greatest in the absence of ZMPSTE24, less when ZMPSTE24 was expressed at the endogenous level, and the least upon ZMPSTE24 overexpression.

### ZMPSTE24 is a restriction factor for a live virus infection with the coronavirus mouse hepatitis virus.

**FIG 6:**
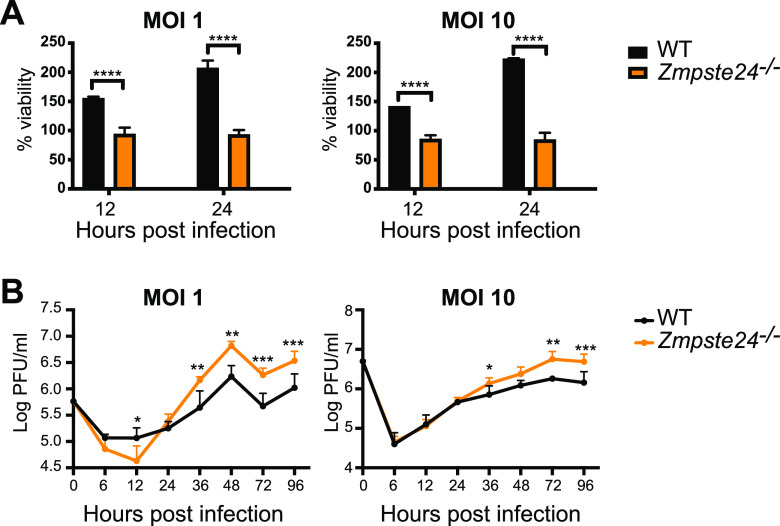
Replication of MHV is enhanced in MEFs from *Zmpste24^−/−^* versus WT mice. MEFs were infected with MHV-A59 at MOIs of 1 and 10. (A) Cell viability was determined by trypan blue exclusion. The data presented are the number of viable cells at 12 and 24 h compared to day 0 (D0), expressed as the percentage of D0 cells. (B) Virus production was measured by assaying supernatant fluids for plaque formation in L929 cells. We note that up to 24 h postinfection we observed a decrease in viability, as shown in panel A, but after this, cells began to grow despite the viral production indicated here. The data are presented as the mean ± SD obtained from three independent experiments. Significance was determined by two-way analysis of variance with Tukey’s multiple-comparison test. ns, *P* > 0.05; *, *P* < 0.05; **, *P* < 0.01; ***, *P* < 0.001; ****, *P* < 0.0001.

Up to this point, our conclusion that ZMPSTE24 can act as a coronavirus restriction factor was based on SARS-CoV-2 Spike-mediated pseudovirus or cell fusion assays, which may not reflect a true live viral infection. To determine if ZMPSTE24 protects cells from a live coronavirus infection, we examined infection of mouse cells by MHV, a murine betacoronavirus distantly related to SARS-CoV-2 but that is amenable to testing at a biosafety level 2 (BSL-2) laboratory (52, 53). The MHV Spike glycoprotein binds a cell surface receptor (CEACAM1a) that is widely expressed in mammalian cells. WT and *Zmpste24^−/−^* mouse embryonic fibroblasts (MEFs) were infected with MHV at a multiplicities of infection (MOIs) of 1 and 10, and cell viability and plaque assays were performed (Fig. 6). At 12 and 24 h postinfection, *Zmpste24^−/−^* MEFs were significantly less viable than their WT counterparts, as assayed by trypan blue exclusion (Fig. 6A), indicating a protective role for endogenous ZMPSTE24 against loss of viability caused by MHV infection. Furthermore, viral replication, as assayed by PFU between 24 and 96 h postinfection, was significantly enhanced in *Zmpste24^−/−^* versus WT MEFs, at both MOIs tested (Fig. 6B). These results reinforced the conclusion that endogenous ZMPSTE24 is a viral restriction factor that protects cells against infection, for at least one type of coronavirus.

## DISCUSSION

ZMPSTE24 has been previously shown to defend cells against entry of many, but not all, enveloped viruses (5, 29, 30, 54, 55). In the present study, we have extended the range of viruses against which ZMPSTE24 protects to include coronaviruses. We show here that ZMPSTE24 protects cells against SARS-CoV-2 Spike-mediated pseudovirus infection and syncytia formation. Specifically, cells in which ZMPSTE24 is knocked out or its expression knocked down are sensitized to infection by SARS-CoV-2-pseudotyped lentivirus, compared to WT cells (Fig. 2). Furthermore, overexpression of ZMPSTE24 restricts SARS-CoV-2 pseudovirus infection to a similar extent as overexpression of IFITMs1-3 (Fig. 3) (44, 45). In addition, we demonstrated that like IFITM1-3 (45, 49, 51), ZMPSTE24 modulates SARS-CoV-2 Spike-ACE2-mediated cell-to-cell fusion in a dose-dependent manner (Fig. 4 and 5). ZMPSTE24 knockout or knockdown cells were sensitized to fusion compared to WT cells, while overexpression of ZMPSTE24 dampened fusion. Thus, ZMPSTE24, like the IFITM proteins, may be an important component of the innate immune response to protect against a growing list of enveloped pathogenic viruses.

The exact mechanism of ZMPSTE24-mediated protection against enveloped viruses remains to be elucidated. The best-characterized role for ZMPSTE24 is the proteolytic maturation of the nuclear lamin A precursor, prelamin A, in mammalian cells (15, 56, 57) and the a-factor mating pheromone in yeast (27, 28). More recently, ZMPSTE24 and its yeast homolog Ste24 have also been implicated in ER quality control pathways, including the clearance of clogged translocons and prevention of improper targeting of proteins to the ER (21–25). In addition, yeast cells require Ste24 for proper insertion of a membrane topology reporter protein (26). To perform all of these functions, ZMPSTE24 must be catalytically active. In contrast, the catalytic activity of ZMPSTE24 is dispensable in its role of viral restriction. Z*mpste24^−/−^* MEFs transduced with human ZMPSTE24 or a catalytically inactive variant (with an E-to-A change at position 336 [E336A]) displayed equal protection against the viruses IAV, VSV, and vaccinia virus (VACV) (29). Likewise, transiently overexpressed ZMPSTE24-E336A (or other ZMPSTE24 variants with decreased proteolytic activity) are equally protective as wild-type ZMPSTE24 against IAV or arenaviruses (29, 55). Furthermore, we found that protection from infection by SARS-CoV-2 Spike-pseudotyped virus and diminished Spike-AceIIACE2-mediated cell-to-cell fusion were promoted equally as well as by WT and catalytically dead forms of ZMPSTE24 (Fig. 3 and 5). Examples of enzymes which moonlight to also perform nonenzymatic functions are known. One example is the phosphatase of regenerating liver (PRL), which has catalytic phosphatase activity for certain phosphorylated substrates but also has a non-catalytic role in regulating the integral membrane magnesium transporter CNNM (58, 59). Structural analyses revealed that CNNM mimics a PRL substrate and binds near residues in PRL required for catalysis. Likewise, IFITM3 could bind ZMPSTE24 in a manner that mimics ZMPSTE24’s bona fide substrate prelamin A. Interestingly, we have shown here that IFITM3 coimmunoprecipitates more efficiently with the catalytically inactive variant ZMPSTE24-E336A than with WT ZMPSTE24 (Fig. 3D). However, overexpression of either version of ZMPSTE24 resulted in essentially equivalent protection from pseudovirus infection and cell-cell fusion (Fig. 3B and 5B). In future studies, it will be of interest to seek specific mutations in either or both ZMPSTE24 and IFITM3 that disrupt their interaction to better dissect the role of their interaction in viral defense.

The observation that ZMPSTE24 and the IFITM proteins interact, and that overexpression of any of these components inhibits pseudovirus infection and cell-to-cell fusion, suggests that ZMPSTE24 and the IFITMs may act at a common step in preventing membrane fusion. The IFITM proteins block viral fusion to host membranes at a stage after outer leaflet lipid mixing, but prior to pore formation (37, 42). Evidence suggests they do so by stiffening membranes through their conserved amphipathic helix, which induces negative curvature (37–40, 42, 50). Interestingly, Dorf and colleagues reported that MEFs knocked out for *Ifitm1-3* that overexpressed human ZMPSTE24 were resistant to infection by multiple viruses, but MEFs knocked out for *Zmpste24* that overexpressed IFITM3 were sensitive to viral infection (29). These data suggested that IFITMs may require ZMPSTE24 for their antiviral activity, but that ZMPSTE24 can prevent infection independently of the IFITM proteins (although this was tested for only a few viruses [29, 30] and may not be a ubiquitous mechanism). A recent report found that IFITMs act cooperatively with ZMPSTE24 to restrict arenavirus infection (55). One possibility is that ZMPSTE24 controls membrane fluidity by directly or indirectly altering lipid content in the cell, which could then affect IFITM accumulation or activity at the sites of viral-host cell membrane fusion. Alternatively, ZMPSTE24 could bind cholesterol, as IFITM3 has been shown to do (60, 61), either on its own or together with the IFITMs, in a way that contributes to blocking viral host-cell fusion. Further studies examining whether ZMPSTE24 affects membrane composition, and/or whether loss of ZMPSTE24 prevents membrane stiffening by the IFITM proteins, could help explain the interdependence of the IFITMs and ZMPSTE24.

Although ZMPSTE24 has been localized to the ER and INM (20), it is unknown whether ZMPSTE24 can be recruited to other cellular sites. IFITM1 localizes mostly to the plasma membrane, whereas IFITM2 and IFITM3 are predominantly on endolysosomal membranes (6, 31, 34, 41, 62). A possibility is that when IFITMs are present, due to interferon induction or artificial overexpression, they can recruit some or all of ZMPSTE24 to assist in blocking viral fusion. Alternatively, ER-localized ZMPSTE24 may act directly on the IFITM proteins to ensure their proper topology, localization, stability, or a posttranslational modification required for their activity (i.e., palmitoylation [63, 64]). Both models are consistent with the findings of Dorf and coworkers that IFITM3 overexpression in *Zmpste24^−/−^* MEFs was not protective against IAV, VSV, and VACV (29). We note that care must be taken in making conclusions about ZMPSTE24 localization when using tagged constructs. ZMPSTE24 has a C-terminal ER KxxKxx retrieval motif (KTMKQH), and we have shown that addition of a C-terminal epitope tag disrupts this signal, causing aberrant localization of ZMPSTE24 in a Golgi/endolysosmal compartment, whereas an N-terminally tagged construct is properly localized to the ER/INM (20) (see also Fig. S1 in the supplemental material). Therefore, conclusions about ZMPSTE24 localization in studies which use C-terminally tagged ZMPSTE24 constructs must be viewed with caution (29, 55). Careful studies on the localization of ZMPSTE24 and the IFITMs when expressed individually and together and/or in the context of viral or pseudoviral infection will be critical for understanding whether relocalization of ZMPSTE24 from the ER/INM plays a role in its antiviral function.

10.1128/mbio.02543-22.1 FIG S1 C-terminal tagging of ZMPSTE24 disrupts its ER retrieval motif and normal ER/INM localization, causing aberrant localization in a Golgi/endosomal compartment. N- or C-terminally Flag-tagged ZMPSTE24 proteins were transiently expressed in HeLa cells. Their subcellular localization was determined by immunofluorescence using anti-FLAG antibodies (green) and costaining for the indicated organelle markers (red), as follows: ER (calnexin) (A); Golgi (GM130) (B); endosomes (EEA1) (C). Nuclei were visualized by DAPI staining (blue). ZMPSTE24 C-terminal tagging (right panels) induced abnormal localization of ZMPSTE24 in Golgi/endosomal compartments instead of its standard location in the ER membrane and INM (20), as seen for N-terminally tagged ZMPSTE24 (left panels). Download FIG S1, TIF file, 2.7 MB. Copyright © 2022 Shilagardi et al. 2022 Shilagardi et al. https://creativecommons.org/licenses/by/4.0/ This content is distributed under the terms of the Creative Commons Attribution 4.0 International license.

In addition to demonstrating that human cells in which ZMPSTE24 is knocked out, or its expression knocked down, are sensitized to SARS-CoV-2 pseudovirus infection (Fig. 2), we have also provided evidence that mouse *Zmpste24^−/−^* knockout fibroblasts are more sensitive than WT fibroblasts to a bona fide viral infection, using the mouse betacoronavirus MHV and assaying viral production by plaque assay as well as cell viability upon infection (Fig. 6). This finding amplified our point that ZMPSTE24 may be broadly protective against coronaviruses. However, in this regard it is important to note that the simple picture that IFITMs exclusively function as antiviral factors has been complicated by recent studies suggesting that rather than defending against infectivity, IFITMs can actually be essential host factors, required for live virus infection for certain viruses (44–46). Thus, it will be important to assess whether ZMPSTE24 acts as an antiviral factor in a true SARS-CoV-2 infection assay; these experiments will require testing under BSL-3 conditions.

Understanding the mechanism of how ZMP functions as a viral restriction factor either on its own, together with IFITMs, or possibly with other cellular factors could provide the basis for developing a novel antiviral therapeutic target against existing or emergent viruses. In addition, some evidence points to diminished expression of ZMPSTE24 as a feature in normal aging (65). Thus, it is tempting speculate that a low level of ZMPSTE24 could contribute to increased vulnerability to viral infection in aging individuals.

## MATERIALS AND METHODS

### Plasmids.

Plamsids used in this study are listed in Table 1. Plasmid pSM3938 expressing Sars-CoV-2 Spike protein was used to produce Spike-pseudotyped lentivirus. It was constructed by PCR mutagenesis and recombinational cloning using pSM3878 as a template. pSM4020 expressing VSV-G was used to produce VSV-G-pseudotyped lentivirus. It was constructed by replacing the SARS-CoV-2 Spike sequence in pSM3938 with a PCR-amplified VSV-G sequence from plasmid pSM4019/pMD2.G (a gift from Peter Espenshade, Johns Hopkins School of Medicine). Plasmids expressing Myc-IFITM1 and Myc-IFITM2 were constructed by subcloning EcoRI-SalI fragments containing IFITM1 and IFITM2 genes from pSM3865 and pSM3866 to replace hIFITM3 in pSM3867. pSM3914 contains 2XFlag-tagged ZMPSTE24 and was constructed by PCR amplifying Flag-ZMPSTE24 from pSM3283 (16) and replacing Myc-IFITM3 in pSM3867. 2XFlag-ZMPSTE24-E336A (pSM3915) was generated from this by PCR mutagenesis of pSM3914. Sequences of plasmids used in this study are available upon request.

**TABLE 1:** Plasmids used in this study

pSM	Relevant genotype or protein	Source
pSM3865	P*_CMV_-*HA-hIFITM1	Howard Hang, Jacob Yount; Addgene 58399
pSM3866	P*_CMV_-*HA-hIFITM2	Howard Hang, Jacob Yount; Addgene 58398
pSM3867	P*_CMV_-*Myc-hIFITM3	Jacob Yount; Addgene 58461
pSM3873	P*_CMV_-*Myc-hIFITM1	This study
pSM3874	P*_CMV_-*Myc-hIFITM2	This study
pSM3878	HDM_SARS2_SpikeΔ21_D614G	Jesse Bloom; Addgene 158762
pSM3900	pHAGE-CMV-Luc2-IRES-ZsGreen-W	BEI Resources NR-52516
pSM3901	HDM-Hgpm2	BEI Resources NR-52517
pSM3902	HDM-tat1b	BEI Resources NR-52518
pSM3903	pRC-CMV-Rev1b	BEI Resources NR-52519
pSM3914	P*_CMV_-*2XFlag-ZMPSTE24	This study
pSM3915	P*_CMV_-*2XFlag-ZMPSTE24-E336A	This study
pSM3938	HDM_SARS2_SpikeΔ21_N501Y, D614G	This study
pSM4018	ITRSB-ACE2-mCh-2a DHFR(DD)-puro	Steve Gould; Johns Hopkins School of Medicine
pSM4019	P*_CMV_-*VSV-G	Peter Espenshade; Johns Hopkins School of Medicine
pSM4020	HDM_VSV-G	This study

### Cell lines.

Cells were grown in Dulbecco’s modified Eagle’s medium (DMEM) with high glucose without l-glutamine, supplemented with 10% fetal bovine serum and 1× penicillin-streptomycin-glutamine. CRISPR/Cas9-mediated ZMPSTE24 knockout (ZMSTE24-KO) HeLa cells (provided by Fabio Martinon, University of Lausanne) were previously described (66). HEK293T-ACE2 cells were obtained through BEI Resources (catalog number NR-52511). ZMPSTE24-KO and WT HEK293T-ACE2-mCherry stable clonal cell lines were created by fluorescence-activated cell sorting followed by limited dilution of ZMPSTE24-KO and WT HEK293T cells (provided by Alex Compton, NCI) transformed with pSM4018. WT and *Zmpste24^−/−^* MEFs (8, 67) were obtained from Stephen Young (UCLA). L-929 cells, a mouse fibroblast cell line, were obtained from ATCC (catalog number CCL-1).

### Generation of pseudotyped lentiviral particles.

Pseudotyped lentiviruses were generated, titrated, and stored essentially as described previously (68), with minor modifications. Briefly, HEK293T cells were seeded into 6-well plates so they would reach ~60% confluence the following day. At 16 h after seeding, cells were transfected using Lipofectamine 3000 (Thermo Fisher) with the following plasmid cocktail: 1.25 μg of lentiviral backbone vector luciferase-IRES-ZsGreen, 0.25 μg each of helper plasmids HDM-Hgpm2, pRC-CMV-Rev1b, and HDM-tat1b, and 0.5 μg of plasmid expressing viral entry protein, either pSM3938 or pHDM-VSV-G, for the production of SARS-CoV-2 or VSV-G pseudovirus, respectively. At 65 h posttransfection, the supernatant from each well was collected, pooled, centrifuged at 500 × *g*, and filtered through a 0.45-μm low-protein-binding filter. The resulting virus suspension was aliquoted and frozen at −80°C for future experiments. Pseudovirus titers were determined according to the methods of Crawford et al. (68). Briefly, either HEK293T cells (VSV-G-pseudotyped virus) or HEK293T-ACE2 cells (Spike-pseudotyped virus) were used as target cells, and infection efficiency was monitored by expression of ZsGreen reporter in the infected cell population. Infection resulting in 10% of ZsGreen positivity in the population reflected an MOI of 0.1.

### Pseudovirus infection assay.

For infection experiments, cells were seeded to a density of ~0.5 × 10^5^ cells per well in 24-well plates. After 16 h, cells were infected with VSV-G- or Spike-pseudotyped lentivirus at an MOI of 0.1 in the presence of 5 μg/mL Polybrene (Sigma-Aldrich). Three separate wells were designated for each experimental condition to achieve biological triplicates. At 48 h postinfection, cells were trypsinized and counted, and 1.5 × 10^4^ cells/well in 100 μL of growth medium were added into each well of 96-well plate. The cells were lysed by adding 100 μL of luciferase assay reagent (Bright-Glo Luciferase Assay System, Promega) and incubated for 2 min at room temperature in the dark, and then luminescence was measured using a fluorescence plate reader with a luminescence integration time of 1 s. Data were analyzed using Omega data analysis software. To visualize infection using ZsGreen expression, ~0.5 × 10^5^ cells per well were seeded in 24-well plates containing glass coverslips and infected as described above. At 48 h postinfection, cells on the coverslips were fixed and processed for confocal microscopy (see below).

### Cell-to-cell fusion assay.

Cell fusion was achieved by mixing equal amounts of “donor” cells (expressing Spike protein) and “recipient” cells (expressing hACE2 receptor). The cell populations were mixed and seeded into individual wells of a 24-well plate equipped with glass coverslips. Three separate wells were designated for each experimental condition. The cell fusion efficiency was assessed 3 h postmixing by processing cells on the coverslips for confocal microscopy and counting nuclei in syncytia versus the total number of nuclei on a given microscopic field. The donor cell population was identical throughout the given experimental setup, where effects of overexpression of certain proteins were directly compared (for example, for all the experiments shown in Fig. 5).

### siRNA-mediated gene silencing.

For siRNA-mediated knockdown of ZMPSTE24, HEK293T-ACE2 cells were seeded in 24-well plates at a density of ~0.6 × 10^5^ cells per well. After 16 h, cells were transfected with scrambled control siRNA (Silencer Select, negative control#1; Ambion catalog number 4390843) or siRNA targeting ZMPSTE24 (Silencer Select, validated siRNA, Ambion catalog number 4427038) using Lipofectamine RNAiMAX reagent (Thermo Fisher). At 36 h posttransfection, cells were used either for infection or cell fusion assays.

### Transient transfection experiments.

To transiently overexpress ZMPSTE24 or IFITM proteins, HEK293T-ACE2 or HEK293T-ACE2-mCherry cells were seeded in 24-well plates at a density of ~0.6 × 10^5^ cells per well and 16 h later were transfected with plasmids overexpressing either ZMPSTE24 or IFITM proteins using Effectene (Qiagen). At 36 h posttransfection, cells were used for either infection or cell fusion assays.

To create the donor cell population, HEK293T cells were seeded in 6-well plates at a density of ~2.5 × 10^5^ cells per well and 16 h later were transfected with pSM3938. At 36 h posttransfection, cells were lifted by trypsinization and used for cell fusion assays.

### Preparation of lysates, SDS-PAGE, and Western blot analysis.

Cells in individual 24-well plates were harvested by direct, “in-well” lysis using SDS loading buffer and heated at 65°C for 10 min prior to SDS-PAGE. Lysates from ~1 × 10^4^ cells were resolved by 4 to 20% SDS-PAGE and transferred onto a nitrocellulose membrane. The membrane was blocked using 5% nonfat dry milk in phosphate-buffered saline containing 0.1% Tween 20 (PBST) for 1 h at room temperature. Blots were probed overnight at 4°C with primary antibodies, washed extensively with PBST, and incubated with the appropriate secondary antibodies for 30 min. Blots were washed again with PBST (3 times, 10 min each), and signals were detected on the Odyssey CLx imaging system using Image Studio Lite software (LI-COR). Antibodies used are listed in Table 2.

**TABLE 2:** Antibodies used in this study

Antibody	Dilution	Source
Primary antibodies		
Mouse anti-FLAG	1:5,000	Sigma F3164
Goat anti-ACE2	1:1,000	R&D Systems AF933SP
Mouse anti-β-actin	1:2,000	Santa Cruz Biotechnology sc-47778
Rabbit anti-ZMPSTE24	1:500	Thermofisher 16965
Rat anti-prelamin A (3C8)	1:2,000	Stephen Young, UCLA
Secondary antibodies		
Donkey anti-mouse IRDye 680RD	1:15,000	LI-COR Biosciences
Goat anti-rat IRDye680RD	1:15,000	LI-COR Biosciences
Goat anti-rabbit IRDye 800CW	1:15,000	LI-COR Biosciences
Donkey anti-goat IRDye 800CW	1:15,000	LI-COR Biosciences

### Coimmunoprecipitation.

HEK293T cells (~0.3 × 10^6^) were transiently transfected with 100 ng of Myc-IFITM3 and 100 ng of vector, Flag-ZMPSTE24, or Flag-ZMPSTE24-E336A. After ~40 h, cells were collected, washed in cold PBS, and lysed in 50 mM HEPES (pH 7.5), 150 mM NaCl, 1% Triton X-100, 0.5 mM phenylmethylsulfonyl fluoride and 1× protease inhibitor cocktail (Roche). Proteins were solubilized by mixing the lysate end-over-end at 4°C for 1 h and subsequently centrifuged at 100,000 × *g* for 1 h to yield a soluble input fraction. Proteins were immunoprecipitated using 10 μL anti-FLAG M2 magnetic beads (Sigma) for 2 h at 4°C. The beads were washed twice with lysis buffer, then once with lysis buffer without detergent. Proteins were eluted from beads by incubating in 1× SDS sample buffer without reducing agent for 15 min at 65°C and were analyzed by SDS-PAGE and Western blotting as described above.

### Fixation of cells and confocal microscopy analysis.

Fixation and staining procedures were performed at room temperature, and the amounts of reagents described are for a single coverslip in one well of a 24-well plate. Cells on coverslips in each well were fixed in 0.2 mL of 4% freshly prepared paraformaldehyde for 20 min and rinsed 3 times with 0.2 mL PBS. Fixed cells were permeabilized by incubating in 0.2 mL of PBS with 0.1% Triton X-100 for 10 min, followed by rinsing once with 0.2 mL PBS–Triton X-100 and 2 times with 0.2 mL PBS, and the fixed cells were stained with 5 μg/mL 4′,6-diamidino-2-phenylindole (DAPI) solution in PBS for 10 min. DAPI solution was removed and the plates were rinsed 3 times with 0.2 mL PBS. The coverslips with cells were dried and mounted on microscope slides using ProLong Gold Antifade mounting solution (Thermo Fisher Scientific). Images were acquired by Zeiss LSM confocal microscope using an EC Plan-Neofluar 40×, 1.30 numerical aperture differential interference contrast oil immersion objective at a single focal plane. For cell fusion assays, five random 40× microscopic fields from 3 separate coverslips were imaged (*n* = 15), and the total nuclei count on each field ranged from 54 to 87. Multinucleated cells with >2 nuclei were counted as a syncytium. Images were processed using Zen Black Edition software using CZI format files. Cell fusion efficiency was expressed as the percentage of the nuclei in syncytia versus total number of nuclei on the given microscopic field.

### Virus infection and quantification of replication by plaque assay.

MHV-A59 was obtained from Matthew Frieman (University of Maryland School of Medicine, Baltimore, MD) and grown and titrated in L929 cells. MEFs were infected at MOIs of 1 and 10 in DMEM with 1% FBS, followed by incubation at 37°C. Virus production was measured by plaque assay on L929 cells. In brief, to quantify virus production, supernatant fluids were collected and titers were determined for plaque formation on L929 cells overlaid with 1.2% Bacto agar in 2× minimal essential medium, after incubation at 37°C for 48 h in a 5% CO_2_ incubator. Cells were fixed with 10% formalin in PBS and stained with 1% crystal violet in 20% ethanol, and plaques were counted. Data are plotted as the means of the log_10_ values of PFU ± standard deviation (SD). Cell viability was determined by trypan blue exclusion assay and is presented as percent day 0 cells.

### Data availability.

All relevant data are provided within the paper and its supporting information files.
