# Supplementary figures and images for: The Integral Membrane Protein ZMPSTE24 Protects Cells from SARS-CoV-2 Spike-Mediated Pseudovirus Infection and Syncytia Formation
Source: mBio. 2022 Oct 5;13(5):e02543-22. doi: 10.1128/mbio.02543-22 (PMC9601121; doi:10.1128/mbio.02543-22)

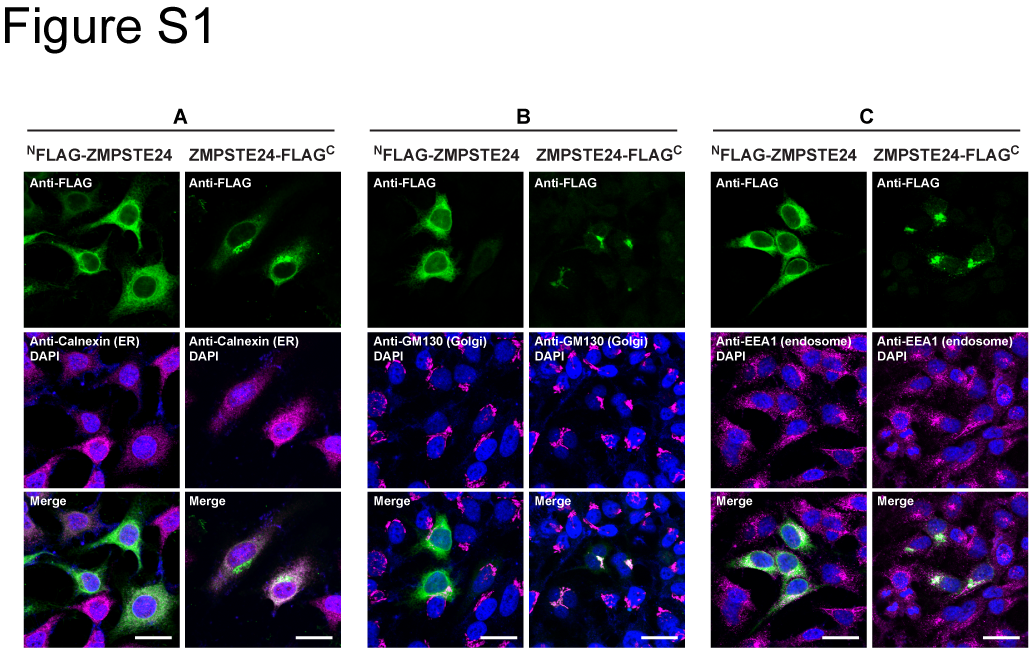

Supplement: FIG S1 [file mbio.02543-22-s0001.tif]
